# Association between TGFBR1 Polymorphisms and Cancer Risk: A Meta-Analysis of 35 Case-Control Studies

**DOI:** 10.1371/journal.pone.0042899

**Published:** 2012-08-08

**Authors:** Yong-qiang Wang, Xiao-wei Qi, Fan Wang, Jun Jiang, Qiao-nan Guo

**Affiliations:** 1 Institute of Pathology, Southwest Hospital, Third Military Medical University, Chongqing, China; 2 Breast Disease Center, Southwest Hospital, Third Military Medical University, Chongqing, China; 3 Department of Oncology, Jiangjin Central Hospital, Jiangjin, Chongqing, China; University of Porto, Portugal

## Abstract

**Background:**

Numerous epidemiological studies have evaluated the association between TGFBR1 polymorphisms and the risk of cancer, however, the results remain inconclusive. To derive a more precise estimation of the relation, we conducted a comprehensive meta-analysis of all available case-control studies relating the TGFBR1*6A and IVS7+24G>A polymorphisms of the TGFBR1 gene to the risk of cancer.

**Methods:**

Eligible studies were identified by search of electronic databases. Overall and subgroup analyses were performed. Odds ratio (OR) and 95% confidence interval (CI) were applied to assess the associations between TGFBR1*6A and IVS7+24G>A polymorphisms and cancer risk.

**Results:**

A total of 35 studies were identified, 32 with 19,767 cases and 18,516 controls for TGFBR1*6A polymorphism and 12 with 4,195 cases and 4,383 controls for IVS7+24G>A polymorphism. For TGFBR1*6A, significantly elevated cancer risk was found in all genetic models (dominant OR = 1.11, 95% CI = 1.04∼1.18; recessive: OR = 1.36, 95% CI = 1.11∼1.66; additive: OR = 1.13, 95% CI = 1.05∼1.20). In subgroup analysis based on cancer type, increased cancer risk was found in ovarian and breast cancer. For IVS7+24G>A, significant correlation with overall cancer risk (dominant: OR = 1.39, 95% CI = 1.15∼1.67; recessive: OR = 2.23, 95% CI = 1.26∼3.92; additive: OR = 1.43, 95% CI = 1.14∼1.80) was found, especially in Asian population. In the subgroup analysis stratified by cancer type, significant association was found in breast and colorectal cancer.

**Conclusions:**

Our investigations demonstrate that TGFBR1*6A and IVS7+24G>A polymorphisms of TGFBR1 are associated with the susceptibility of cancer, and further functional research should be performed to explain the inconsistent results in different ethnicities and cancer types.

## Introduction

Cancer is a disease resulting from complex interactions between environmental and genetic factors [1]–[3]. Genetic factors, including the sequence alterations and organization aberrations of the cellular genome that range from single-nucleotide substitutions to gross chromosome, could modulate several important biological progress and alert susceptibility to cancer consequently.

The transforming growth factor-β (TGF-β) signaling pathway has been the focus of extensive research since it was first discovered in 1981 [4], [5]. It has now been well established that this signaling pathway is an important modulator of several biological processes, including cell proliferation, differentiation, migration and apoptosis [6]. Aberrations of the TGF-β signaling pathway are frequently found in many diseases including human cancers in breast, colon, prostate or pancreas [7]–[10]. As overall TGF-β signaling may be determined by genetic polymorphisms in several TGF-β pathway genes, an increasing number of studies have pointed to the effects of TGF-β pathway gene variants on cancer risk. As the central propagator of TGF-β signaling pathway, TGF-β receptor type I (TGFBR1) has been the hot spot of research.

TGFBR1 gene locates on chromosome 9q22 [11]. Two commonly studied polymorphisms of TGFBR1 gene are TGFBR1*6A (rs1466445), which results from the deletion of three alanines within a nine-alanine (*9A) stretch in exon 1 [12] and IVS7+24G>A (rs334354), which represents a G to A transversion in the +24 position of the donor splice site in intron 7. Although the functional role of IVS7+24G>A is unclear yet, TGFBR1*6A has been suggested to be responsible for efficiency in mediating TGF-β growth inhibitory signals [13]. Therefore, it is biologically reasonable to hypothesize that polymorphisms of TGFBR1 gene may play a functional role in carcinogenesis.

A number of studies have investigated the association between TGFBR1 polymorphisms and cancer risk, but results are somewhat controversial and underpowered. For TGFBR1*6A, a recent meta-analysis in 2010 by Liao et al. [14] found significant association with overall cancer, however, several new papers are further available [15]–[23]. With respect to IVS7+24G>A polymorphism, only 2 meta-analysis on this issue had ever appeared [24], [25]. Zhang [24] found the IVS7+24G>A carriers had a 76% increase of risk of cancer (OR = 1.76, 95% CI = 1.33∼2.34) with only 440 cases and 706 controls in 3 studies. Meanwhile, Zhang et al. [25] limited the investigation on colorectal cancer and found that there was a significantly increased risk for homozygosity A/A carriers compared to heterozygosity and homozygosity of the allele G carriers (OR = 1.71, 95% CI = 1.17∼2.51). To derive a more precise estimation of the relationship between TGFBR1 polymorphisms and cancer risk, we carried out an updated meta-analysis of all available case–control studies relating the TGFBR1*6A and/or IVS7+24G>A polymorphisms of the TGFBR1 gene to the risk of cancer. To the best of our knowledge, this is the most comprehensive meta-analysis regarding the TGFBR1 polymorphisms and cancer risk.

**Figure 1:**
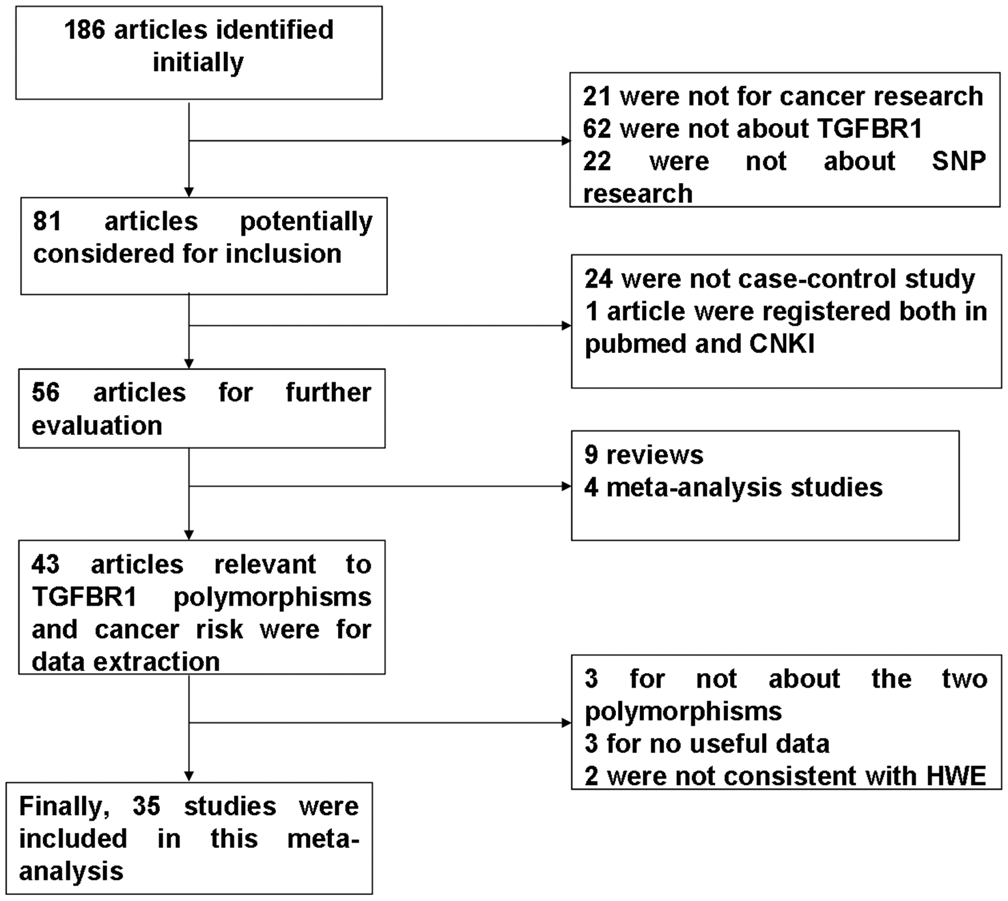
Flow diagram of study identification.

**Table 1:** Characteristics of case-control studies included in TGFBR1 TGFBR1*6A polymorphism and cancer risk.

First author	Year	Country	Ethnicity	Cancer type	Sample size	Case			Control		
					**(case/control)**	**9A/9A**	**6A/9A**	**6A/6A**	**9A/9A**	**6A/9A**	**6A/6A**
Pasche [36]	1999	USA	Mixed	Colon	111/732	90	17	4	654	78	0
Pasche [36]	1999	USA	Mixed	Ovarian	47/732	39	7	1	654	78	0
Pasche [36]	1999	USA	Mixed	Breast	152/732	128	24	0	654	78	0
Pasche [36]	1999	USA	Mixed	Germ cell cancer	56/732	49	5	2	654	78	0
Pasche [36]	1999	USA	Mixed	Lung	94/732	82	11	1	654	78	0
Pasche [36]	1999	USA	Mixed	Prostate	59/732	51	8	0	654	78	0
Pasche [36]	1999	USA	Mixed	Pancreas	14/732	12	2	0	654	78	0
Pasche [36]	1999	USA	Mixed	Bladder	77/732	67	10	0	654	78	0
Pasche [36] a	1999	USA	Mixed	Hematologic	228/732	189	38	1	654	78	0
Pasche [36]	1999	USA	Mixed	Melanoma	10/732	9	1	0	654	78	0
Pasche [36]	1999	Italy	Caucasian	Breast	48/50	39	8	1	38	12	0
Pasche [36]	1999	Italy	Caucasian	Bladder	234/50	199	35	0	38	12	0
Pasche [36]	1999	Italy	Caucasian	Colon	65/50	57	8	0	38	12	0
Chen [37]	1999	USA	NS	Cervical	37/38	29	7	1	34	4	0
Chen [37]	1999	Jamaica	African	Cervical	29/30	26	3	0	27	3	0
van Tilborg [38]	2001	Netherlands	Caucasian	Bladder	146/183	121	25	0	148	32	3
Stefanovska [39]	2001	Macedonia	Caucasian	Colorectal	117/200	108	8	1	179	20	1
Samowitz [40]	2001	USA	Mixed	Colon	250/358	202	46	2	295	58	5
Baxter [41]	2002	UK	Caucasian	Breast	355/248	268	83	4	207	39	2
Baxter [41]	2002	UK	Caucasian	Ovarian	304/248	236	62	6	207	39	2
Chen [12]	2004	USA	Mixed	Renal	88/138	71	15	2	112	25	1
Chen [12]	2004	USA	Mixed	Bladder	63/138	49	13	1	112	25	1
Kaklamani [42]	2004	USA	Mixed	Prostate	442/465	380	59	3	402	62	1
Reiss [43]	2004	USA	Mixed	Breast	98/91	87	11	0	77	14	0
Ellis [43]	2004	USA	Ashkenazi Jews	Colon	767/766	655	108	4	663	100	3
Caldes [43]	2004	Spain	Caucasian	Breast	271/292	214	56	1	250	42	0
Caldes [43]	2004	Spain	Caucasian	Colorectal	235/292	183	50	2	250	42	0
Offit [43]	2004	USA	NS	Breast	462/330	391	67	4	291	38	1
Northwestern [43]	2004	USA	NS	Breast, Ovarian	86/123	74	12	0	105	17	1
Northwestern [43]	2004	USA	NS	Colon	35/123	30	5	0	105	17	1
Jin [44]	2004	Finland	Caucasian	Breast	221/234	177	38	6	171	60	3
Jin [44]	2004	Poland	Caucasian	Breast	170/202	140	28	2	176	26	0
Suarez [45]	2005	USA	Mixed	Prostate	534/488	441	87	6	407	79	2
Spillman [46]	2005	USA	Mixed	Ovarian	578/607	468	100	10	497	104	6
Kaklamani [47]	2005	USA	Mixed	Breast	611/690	515	92	4	612	77	1
Chen [48]	2006	USA	Mixed	Breast	115/130	92	23	0	111	18	1
Feigelson [49] b	2006	USA	Mixed	Breast	481/484	387	94		384	100	
You [50]	2007	China	Asian	Lung	252/250	217	35	0	219	31	0
Cox [51]	2007	USA	NS	Breast	1187/1673	968	207	12	1352	302	19
Song [52]	2007	Sweden	Caucasian	Breast	763/852	598	152	13	682	160	10
Skoglund [53]	2007	Sweden	Caucasian	Colorectal	1040/852	827	203	10	682	160	10
Skoglund Lundin [54]	2009	Sweden	Caucasian	Colorectal	213/852	167	42	4	682	160	10
Castillejo [55]	2009	Spain	Caucasian	Bladder	1094/1014	887	199	8	812	191	11
Jakubowska [56]	2010	Poland	Caucasian	Breast	318/290	282	33	3	252	38	0
Jakubowska [56]	2010	Poland	Caucasian	Ovarian	144/279	122	22	0	244	35	0
Colleran [57]	2009	Ireland	Caucasian	Breast	960/958	796	154	10	785	160	13
Dai [15]	2009	German	Caucasian	ALL	458/552	390	61	7	456	88	8
Carvajal-Carmona [16]	2010	UK	Caucasian	Colorectal	913/828	746	159	8	673	145	10
Carvajal-Carmona [16]	2010	UK	Caucasian	Colorectal	933/990	772	152	9	843	140	7
Carvajal-Carmona [16]	2010	UK	Caucasian	Colorectal	1152/1333	938	201	13	1119	200	14
Forsti [17]	2010	Sweden	Caucasian	Colorectal	293/558	218	69	6	435	115	8
Hu [18]	2010	China	Asian	Osteosarcoma	168/168	107	51	10	134	31	3
Abuli [19]	2011	Spain	Caucasian	Colorectal	509/513	427	78	4	405	103	5
Dong [20]	2011	China	Asian	Esophageal	482/584	409	69	4	499	79	6
Guo [21]	2011	China	Asian	Gastric	468/584	393	70	5	499	79	6
Joshi [22]	2011	India	Asian	Breast	167/222	163	4	0	213	9	0
Joshi [22]	2011	India	Asian	Breast	42/169	33	8	1	148	19	2
Martinez-Canto [23]	2012	Spain	Caucasian	Colorectal	521/404	442	72	7	334	67	3

a The combination of Leukemia, lymphoma and MM (multiple myeloma).

b This study was excluded from the combined allelic effect and recessive model because of insufficient data on the frequencies of 9A/6A and 6A/6A genotype.

NS: not stated, ALL: acute lymphocytic leukemia.

**Table 2:** Characteristics of case-control studies included in TGFBR1 IVS7+24G>A polymorphism and cancer risk.

First author	Year	Country	Ethnicity	Cancer type	Sample size	Case			Control		
					(case/control)	GG	GA	AA	GG	GA	AA
Chen [37]	1999	USA, Netherlands	Mixed	Cervical	16/38	9	7	0	24	12	2
Chen [12]	2004	USA	Mixed	Renal	86/113	46	36	4	81	32	0
Chen [12]	2004	USA	Mixed	Bladder	65/113	33	28	4	81	32	0
Chen [12]	2006	USA	Mixed	Breast	223/153	120	92	11	113	37	3
Song [52]	2007	Sweden	Caucasian	Breast	767/853	500	238	267	559	265	29
Castillejo [58]	2009	Spain	Caucasian	Colorectal	504/504	296	178	30	333	156	15
Lundin [54]	2009	Sweden	Caucasian	Colorectal	262/856	135	67	12	559	265	29
Zhang [59]	2009	China	Asian	Colorectal	206/838	60	103	43	245	431	162
Dai [15]	2009	German	Caucasian	ALL	456/551	285	147	24	356	170	25
Forsti [17]	2010	Sweden	Caucasian	Colorectal	308/585	220	68	14	382	179	20
Dong [20]	2011	China	Asian	Esophageal	482/584	296	163	23	402	168	14
Guo [21]	2011	China	Asian	Gastric	468/584	291	155	22	402	168	14
Hu [60]	2011	China	Asian	Osteosarcoma	168/168	100	57	11	115	48	5

## Materials and Methods

### Identification and Eligibility of Relevant Studies

This study was performed according to the proposal of Meta-analysis of Observational Studies in Epidemiology group (MOOSE) [26]. A systematic literature search was performed for articles regarding TGFBR1 SNPs associated with cancer risk. The MEDLINE, Embase, and Chinese National Knowledge Infrastructure (CNKI) were used simultaneously, with the combination of terms “TGFBR1 or transforming growth factor receptor 1 or Type I TGF-beta receptor”, “polymorphism or variant or SNP” and “cancer or neoplasm or carcinoma” (up to May 12, 2012). Reference lists of the identified articles were also examined and the literature retrieval was performed in duplication by two independent reviewers (Yong-qiang Wang and Xiao-wei Qi). Studies that were included in the meta-analysis had to meet all of the following criteria: (1) the publication was a case–control study referring to the association between TGFBR1 polymorphisms (TGFBR1*6A and/or IVS7+24G>A) and cancer, (2) the papers must offer the sample size, distribution of alleles, genotypes or other information that can help us infer the results, (3) when multiple publications reported on the same or overlapping data, we used the most recent or largest population as recommended by Little et al. [27], and (4) publication language was confined to English and Chinese.

**Table 3:** Pooled analysis of association of TGFBR1 TGFBR1*6A (rs1466445) and cancer risk.

			Dominant model			Recessive model			Additive model		
			(6A6A+6A9A) VS 9A9A			6A6A VS (6A9A+9A9A)^a^			6A VS 9A		
	N	Case/Control	OR	*P*_h_	*I* ^2^	OR	*P*_h_	*I* ^2^	OR	*P*_h_	*I* ^2^
**Total**	58	19767/18516	**1.105 (1.035**∼**1.181)**	0.024	28.7%	**1.358 (1.113**∼**1.657)**	0.341	6.9%	**1.125 (1.053**∼**1.201)**	0.006	35.1%
**Cancer tpye**											
Colorectal	15	7154/8851	1.076 (0.956∼1.212)	0.048	41.2%	1.222 (0.887∼1.683)	0.523	0.0%	1.085 (0.963∼1.222)	0.016	49.4%
Ovarian	4	1071/1866	1.218 (0.983∼1.510)	0.526	0.0%	**2.296 (1.011**∼**5.218)**	0.160	45.0%	**1.246 (1.022**∼**1.520)**	0.435	0.0%
Breast	17	6421/7647	1.122 (0.978∼1.287)	0.023	45.2%	1.332 (0.921∼1.925)	0.753	0.0%	**1.151 (1.008**∼**1.314)**	0.034	43.1%
Lung	2	346/982	1.173 (0.782∼1.759)	0.861	0.0%	23.503 (0.951∼581.117)			1.203 (0.817∼1.769)	0.697	0.0%
Prostate	3	1035/1685	1.073 (0.848∼1.358)	0.865	0.0%	2.892 (0.780∼10.717)	0.922	0.0%	1.105 (0.885∼1.380)	0.909	0.0%
Bladder	5	1461/2117	0.936 (0.780∼1.122)	0.536	0.0%	0.633 (0.281∼1.426)	0.472	0.0%	0.924 (0.781∼1.095)	0.512	0.0%
Hematologic	2	686/1284	1.185 (0.575∼2.440)	0.007	86.2%	1.331 (0.518∼3.423)	0.197	40.0%	1.197 (0.609∼2.353)	0.007	86.1%
Cervical	2	66/68	1.732 (0.619∼4.849)	0.454	0.0%	3.164 (0.125∼80.193)			1.822 (0.682∼4.862)	0.401	0.0%
**Ethnicity**											
Mixed	20	4108/4183	**1.145 (1.049**∼**1.251)**	0.640	0.0%	**2.908 (1.735**∼**4.877)**	0.072	39.3%	**1.281 (1.149**∼**1.428)**	0.552	0.0%
Caucasian	25	11477/9980	1.037 (0.941∼1.142)	0.011	43.8%	1.159 (0.901∼1.491)	0.901	0.0%	1.045 (0.957∼1.141)	0.017	41.4%
Others	7	2603/2960	**1.208 (1.083**∼**1.347)**	0.668	0.0%	1.086 (0.611∼1.932)	0.876	0.0%	1.038 (0.908∼1.186)	0.529	0.0%
Asian	6	1579/1393	1.272 (0.951∼1.702)	0.089	47.7%	1.489 (0.767∼2.891)	0.403	0.0%	1.265 (0.946∼1.692)	0.052	54.4%
**Publication bias test**											
Begg’s test			*P* = 0.537			*P* = 0.001			*P* = 0.518		
Egger’s test			*P* = 0.256			*P* = 0.000			*P* = 0.129		

*P*_h_: test for heterogeneity, OR: odds ratio, CI: confidence interval, N: number of data sets.

*I^2^* : the percentage of total variation across studies that is a result of heterogeneity rather than chance.

a Random-effects model was used; otherwise, fixed-effects model was used.

**Table 4:** Pooled analysis of association of IVS7+24G>A (rs334354) and cancer risk.

			Dominant model			Recessive model			Additive model		
			(AA+GA) VS GG			AA VS (GA+GG)			A VS G		
	N	Case/Control	OR	*P*_h_	*I* ^2^	OR	*P*_h_	*I* ^2^	OR	*P*_h_	*I* ^2^
**Total**	13	4195/4383	**1.385 (1.146**∼**1.673)**	0.000	75.9%	**2.225 (1.263**∼**3.921)**	0.000	86.0%	**1.432 (1.140**∼**1.798)**	0.000	89.1%
**Cancer type**											
Colorectal	4	1226/2776	1.030 (0.779∼1.362)	0.016	71.0%	**1.379 (1.035**∼**1.837)**	0.354	7.7%	1.081 (0.876∼1.333)	0.025	68.0%
Breast	2	1228/1006	**1.989 (1.673**∼**2.365)**	0.345	0.0%	**5.959 (1.590**∼**22.331)**	0.046	74.9%	**2.536 (2.091**∼**3.076)**	0.256	22.3%
**Ethnicity**											
Caucasian	5	2481/2489	1.194 (0.854∼1.669)	0.000	88.2%	2.282 (0.848∼6.143)	0.000	93.2%	1.310 (0.833∼2.059)	0.000	95.5%
Asian	4	1324/1590	**1.296 (1.116**∼**1.505)**	0.410	0.0%	**1.578 (1.065**∼**2.337)**	0.205	34.6%	**1.267 (1.086**∼**1.478)**	0.190	37.0%
Mixed	4	390/304	**2.283 (1.694**∼**3.082)**	0.820	0.0%	3.481 (0.972∼12.491)	0.292	19.6%	**2.052 (1.580**∼**2.654)**	0.586	0.0%
**Publication bias test**											
Begg’s test			*P = *1.000			*P = *0.246			*P = *0.360		
Egger’s test			*P = *0.867			*P = *0.889			*P = *0.579		

*P*_h_: test for heterogeneity, OR: odds ratio, CI: confidence interval, N: number of data sets.

*I^2^* : the percentage of total variation across studies that is a result of heterogeneity rather than chance.

**Figure 2:**
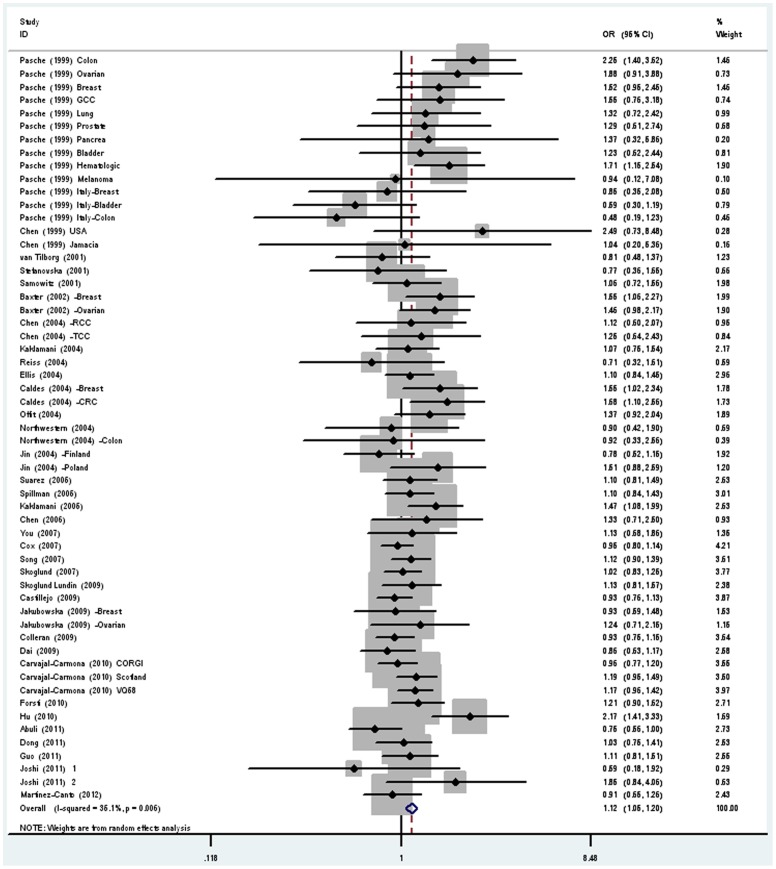
Forest plot (random effects model) describing the association of the TGFBR1*6A polymorphism with risk of cancer. The TGFBR1*6A polymorphism was associated with increased risk of cancer in additive model. Each study is shown by the point estimate of the OR (the size of the square is proportional to the weight of each study) and 95% CI for the OR (extending lines).

### Data Extraction

Two investigators (Yong-qiang Wang and Xiao-wei Qi) independently extracted the data from eligible studies selected according to the pre-specified criteria and the results were compared. Disagreements were resolved by discussion or by involving a third reviewer (Qiao-nan Guo). The following information of each study was collected: first author, reference year, name of studies, total number of cases and controls, studied polymorphisms, ethnicity of subjects, source of controls, and distribution of genotypes in case and control groups. For studies with inadequate information, authors were contacted for further support by E-mail if possible.

**Figure 3:**
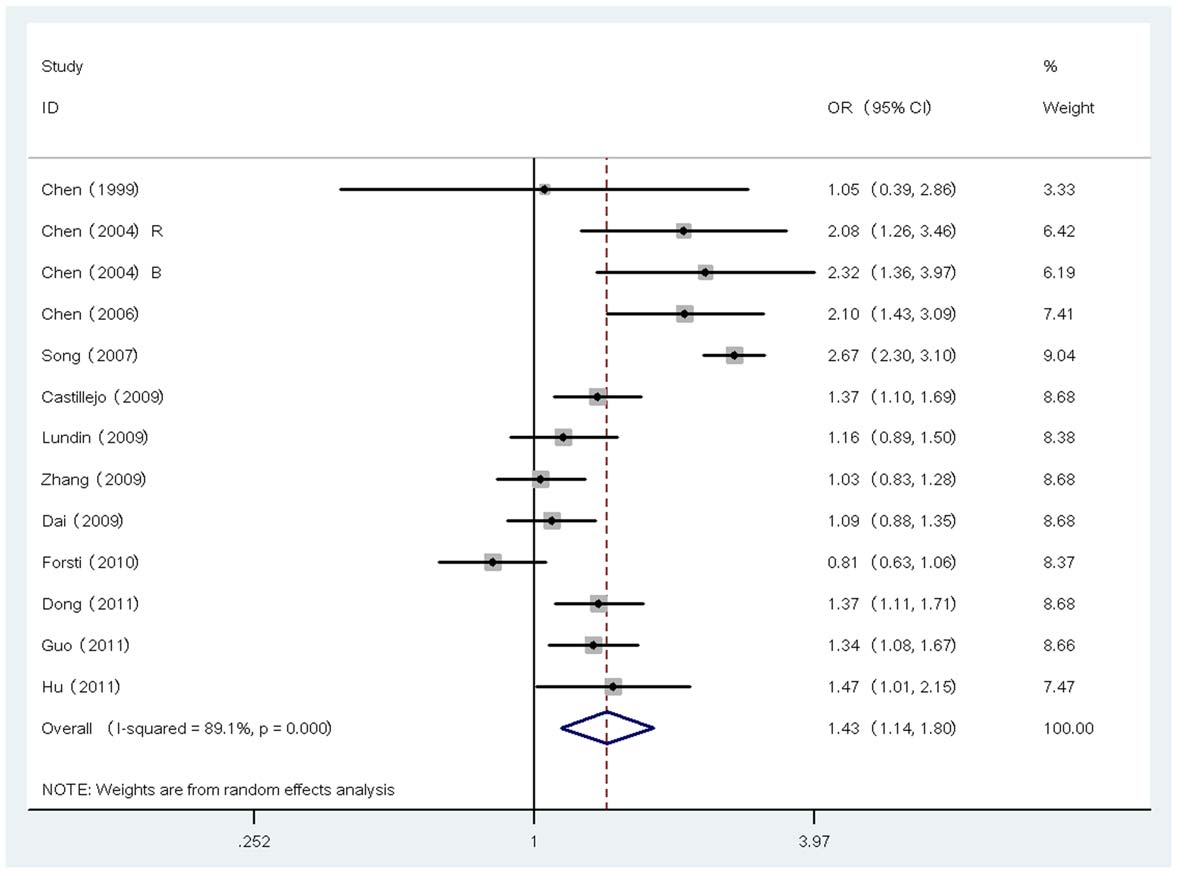
Forest plot (random effects model) describing the association of the IVS7+24G>A polymorphism with risk of cancer. The IVS7+24G>A polymorphism was associated with increased cancer risk in additive model.

### Statistical Analysis

Meta-analysis was performed as described previously [28], [29]. Hardy-Weinberg equilibrium (HWE) in the controls for each study was calculated using goodness-of fit test (chi-square or Fisher’s exact test). It was considered statistically significant when *P*<0.05. Studies deviated from HWE were removed.

**Figure 4:**
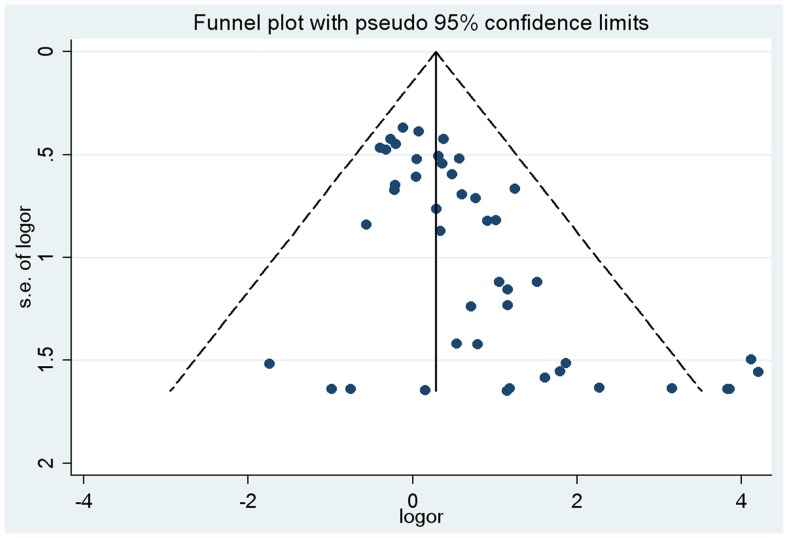
Funnel plot analysis (recessive model of TGFBR1*6A polymorphism) to detect publication bias. Each point represents an individual study for the indicated association. LogOR, natural logarithm of OR. Perpendicular line, mean effect size.

Crude odds ratios (ORs) with their 95% CIs were used to assess the strength of association between polymorphisms of TGFBR1 and cancer risk. The pooled ORs were performed for dominant model (1∶1+1∶2 vs. 2∶2), recessive model (1∶1 vs. 1∶2+2∶2), additive model (1 vs. 2) respectively. 1 and 2 represent the minor and the major allele respectively. Stratified analysis was also performed by ethnicity and cancer type. Leukemia, lymphoma and MM (multiple myeloma) were merged as hematologic cancer. For ethnicity classification, African, Jews and the ethnicity not stated in original study were merged as others.

Heterogeneity assumption was assessed by chi-based Q-test. The heterogeneity was considered statistically significant if *P*<0.10 [30]. With lacking of heterogeneity among studies, the pooled OR was calculated by the fixed effects model (Mantel–Haenszel) [31]. Otherwise, the random effects model (DerSimonian and Laird) was used [32], [33]. We also calculated the quantity *I* ^2^ that represents the percentage of total variation across studies that is a result of heterogeneity rather than chance. Values of less than 25% may be considered “low”, values of about 50% may be considered “moderate”, and values of more than 75% may be considered “high”. A value of 0 (zero) indicates no observed heterogeneity, and larger values show increasing heterogeneity.

**Figure 5:**
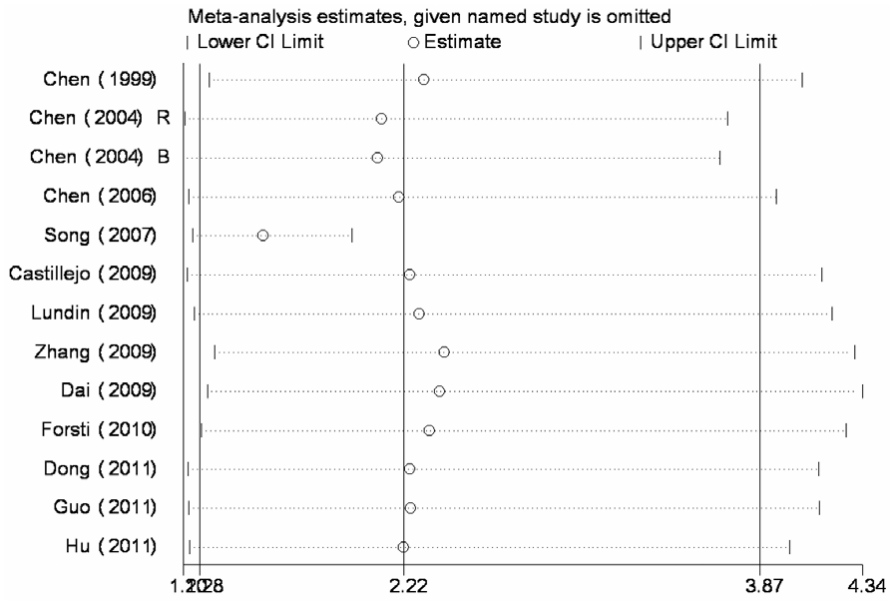
Influence analysis of the summary odds ratio coefficients on the association between IVS7+24G>A polymorphism and cancer risk in recessive model. Results were computed by omitting each study (left column) in turn. Bars, 95% confidence interval.

Sensitivity analysis was carried out by removing each study at a time to evaluate the stability of the results. Publication bias was analyzed by performing funnel plots qualitatively, and estimated by Begg’s and Egger’s test quantitatively [34], [35].

All statistical analysis was conducted using STATA software (version 11.0; STATA Corporation, College Station, TX). Two-sided P-values<0.05 were considered statistically significant.

## Results

### Study Characteristics

After comprehensive searching, a total of 186 publications were identified. We reviewed the titles, abstracts and the full texts of all retrieved articles through defined criteria as shown in **Figure 1**. Finally, the pool of eligible studies included 35 studies [12], [15]–[23], [36]–[60], among which 32 with 19,767 cases and 18,516 controls were for TGFBR1*6A polymorphism and 12 with 4,195 cases and 4,383 controls for IVS7+24G>A polymorphism. Each study in one publication was considered as a data set separately for pooling analysis. **Table 1** and **Table 2** list the main characteristics of these data sets about these two polymorphisms.

### Quantitative Synthesis

The main results of this meta-analysis and the heterogeneity test were shown in **Table 3** and **4**. With respect to TGFBR1*6A polymorphism, a total of 58 data sets in 32 studies were included in this meta-analysis. Of these data sets, 25 were Caucasian, 6 were Asian, 20 were mixed population and 7 were others. Overall, significantly elevated cancer risk was found in all genetic models (dominant model: OR = 1.11, 95% CI = 1.04∼1.18; recessive model: OR = 1.36, 95% CI = 1.11∼1.66; additive model: OR = 1.13, 95% CI = 1.05∼1.20, **Figure 2**). The heterogeneity was significant in all genetic models except for recessive model (*P = *0.34). In the subgroup analysis stratified by ethnicity, significantly increased cancer risk was suggested among mixed ethnicity from US studies (dominant model: OR = 1.15, 95% CI = 1.05∼1.25; recessive model: OR = 1.85, 95% CI = 1.26∼2.72; additive model: OR = 1.22, 95% CI = 1.10∼1.36) but not among Caucasian or Asian population in all genetic models. In the subgroup analysis by cancer type, no significant association with cancer risk was demonstrated in overall population with colorectal, lung, prostate, bladder, hematological and cervical cancer. For ovarian cancer, significantly increased risk was observed in recessive model (OR = 2.30, 95% CI = 1.01∼5.22) and additive model (OR = 1.25, 95% CI = 1.02∼1.52). With respect to breast cancer, significantly increased risk was found only in additive model (OR = 1.15, 95% CI = 1.01∼1.31).

With respect to IVS7+24G>A polymorphism, a total of 12 studies with 13 data sets were included. Of these data sets, 5 were European, 4 were Asian and 4 were from USA with mixed ethnicity. Similar to TGFBR1*6A polymorphism, significantly elevated cancer risk was associated with IVS7+24G>A in all genetic models (dominant model: OR = 1.39, 95% CI = 1.15∼1.67; recessive model: OR = 2.23, 95% CI = 1.26∼3.92; additive model: OR = 1.43, 95% CI = 1.14∼1.80, **Figure 3**). The heterogeneity was significant in all genetic models (*P*<0.1). In the subgroup analysis by ethnicity, significantly increased risk was found in Asian population (dominant model: OR = 1.30, 95% CI = 1.12∼1.51; recessive model: OR = 1.58, 95% CI = 1.07∼2.34; additive model: OR = 1.27, 95% CI = 1.09∼1.48) but not in Caucasian in all genetic models. In the subgroup analysis stratified by cancer type, significantly increased risk was detected in all genetic models in breast cancer (dominant model: OR = 1.99, 95% CI = 1.67∼2.37; recessive model: OR = 5.96, 95% CI = 1.59∼22.33; additive model: OR = 2.54, 95% CI = 2.10∼3.08). With respect to colorectal cancer, significant association was found only in recessive model (OR = 1.38; 95% CI = 1.04∼1.84).

### Publication Bias and Sensitivity Analysis

The shapes of the funnel plots did not reveal any evidence of obvious asymmetry for TGFBR1*6A polymorphism in all genetic models, except for recessive model (**Figure 4**). The Begg’s and Egger’s test also suggested the same results (dominant model: *P_Begg’s_* = 0.54, *P_Egger’s = _*0.26; recessive model: *P_Begg’s_*_ = _0.00 (7.13×10^−4^), *P_Egger’s_* = 0.00(2.23×10^−5^); additive model: *P_Begg’s_* = 0.52, *P_Egger’s = _*0.13). For IVS7+24G>A polymorphism, publication bias was not ruled out not only through visual inspection of asymmetry in funnel plots but also through statistical evidence of the Begg’s and Egger’s test (dominant model: *P_Begg’s_* = 1.00, *P_Egger’s = _*0.87; recessive model: *P_Begg’s_*_ = _0.25, *P_Egger’s_* = 0.89; additive model: *P_Begg’s_* = 0.36, *P_Egger’s = _*0.58).

Sensitivity analysis, which was performed to assess the publication bias and the influence of each individual study on the pooled OR by sequential removal of individual studies, showed that Song’s study [52] was far from the midcourt line for IVS7+24G>A polymorphism in recessive model (**Figure 5**). However, the heterogeneity and the pooled OR were not influenced when this article was excluded (data not shown), which indicated that our results were statistically stable.

## Discussion

In the present study, we explored the association between the TGFBR1*6A and IVS7+24G>A polymorphisms and cancer risk, involving 35 eligible case–control studies. For TGFBR1*6A polymorphism, 19,767 cases and 18,516 controls were included. We found that individuals with the TGFBR1*6A allele showed an increased risk of cancer. In the stratified analysis by cancer type, significantly elevated risks were more pronounced among ovarian cancer and breast cancer. However, no significant correlation of polymorphism TGFBR1*6A with colorectal cancer was found. These findings, though including the latest publications, were consistent with a recent meta-analysis study conducted by Liao et al. [14]. While according to Colleran’s study [57], TGFBR1*6A is not associated with breast cancer. This discrepancy may be due to data missing of some important studies, which was exclusively elaborated by Zhang et al. [61]. Another meta-analysis performed by Zhang et al. [25] found TGFBR1*6A is statistically associated with an increased colorectal cancer risk in dominant model. One factor that may contribute to the differences is that we excluded Castillejo’s study [62] for HWE deviation and included two latest studies [22], [23]. Moreover, a significantly increased risk was found among mixed ethnicity from US studies but not among Caucasian and Asian, and this was the first study evaluating the relation between TGFBR1 polymorphism and overall cancer risk among different populations.

With respect to IVS7+24G>A polymorphism, a previous meta-analysis conducted by Zhang [24] with only 440 cases and 706 controls found that the IVS7+24G>A carriers had a 76% increase of cancer risk. Another meta-analysis conducted by Zhang et al. [25] found that IVS7+24G>A polymorphism had significant effects on colorectal cancer risk in recessive model. However, there were defects in their meta-analysis [25] for mistaking adenoma cases of Lundin’s study [54] as colorectal cancer cases. For the current meta-analysis, 4,195 cases and 4,383 controls were included. Significant correlation of IVS7+24G>A polymorphism with cancer risk was found in all genetic models. When coming to colorectal cancer, the results were in line with Zhang et al [25]. Besides, we also found strong association between IVS7+24G>A polymorphism and breast cancer risk, indicating that potentially functional IVS7+24G>A polymorphism may play a low penetrance role in development of breast cancer. Significant association was found in Asian but not in Caucasian, suggesting a possible role of ethnic differences in genetic backgrounds and the environment they lived in.

To some extent, limitations of this meta-analysis should be addressed. First, the sample sizes of several included studies [12], [37] were rather small and not adequate enough to detect the possible risk for TGFBR1 polymorphisms. Second, cancer is a complex disease with multifactorial etiology. The gene–environment and gene–gene interactions should be further evaluated. Third, haplotype association analysis is the most powerful method to explore the intrinsic effects of gene, but most of the literatures identified in our present meta-analysis were focused on the relation between the two TGFBR1 SNPs and tumor susceptibility, which made it difficult to investigate the TGFBR1 haplotype effects on carcinogenesis. Last but not least, most of US studies were mixed ethnicity, which made it hard to obtain the effects of specific ethnicity on the associations between TGFBR1 polymorphisms and cancer risk.

In summary, this meta-analysis provided evidence that the TGFBR1*9A/6A polymorphism is associated with overall cancer susceptibility and seem to be more susceptible to ovarian and breast cancer. Meanwhile, IVS7+24G>A polymorphism is also associated with increased overall cancer risk especially in colorectal and breast cancer. More well-designed epidemiological studies on specific ethnicity and cancer types, which were not well covered by existing studies, will be necessary to validate the findings identified in the current meta-analysis. Further studies regarding other SNPs (or haplotypes) in the TGFBR1 gene and cancer risk are also encouraged to better understand the role of TGFBR1 in carcinogenesis.
